# Primary Adrenal Lymphoma: Two Case Series From China

**DOI:** 10.3389/fendo.2021.778984

**Published:** 2022-01-28

**Authors:** Jinyang Zeng, Fangfang Yan, Yulong Chen, Li Zang, Kang Chen, Zhaohui Lyu, Jingtao Dou, Yiming Mu, Mingzhu Lin, Guoqing Yang

**Affiliations:** ^1^ Department of Endocrinology and Diabetes, The First Affiliated Hospital of Xiamen University, School of Medicine, Xiamen University, Xiamen, China; ^2^ Department of Endocrinology, Chinese People's Liberation Army (PLA) General Hospital, Beijing, China; ^3^ Department of Endocrinology, Hainan Branch of People's Liberation Army (PLA) General Hospital, Sanya, China

**Keywords:** primary adrenal lymphoma, clinical features, adrenal insufficiency, imaging, histology

## Abstract

**Objective:**

Primary adrenal lymphoma (PAL) is a rare form of adrenal mass. We summarize our experience in its clinical presentation, biochemical indexes, radiological features, pathological information, therapy regimens, and outcomes.

**Methods:**

This was an institutional review board-approved retrospective review of medical records and surgical pathology specimens of patients with a diagnosis of PAL at the Chinese People’s Liberation Army General Hospital and the First Affiliate Hospital of Xiamen University between July 2007 and July 2017.

**Results:**

Twenty-six patients were identified. The mean age at presentation was 60.84 ± 13.14 years with a male-to-female ratio of 2.25:1 (18:8). The most common presenting symptoms were loss of appetite (65%, 17/26), weight loss (62%, 16/26), abdominal pain (58%, 15/26), and fatigue (58%, 15/26). The levels of lactate dehydrogenase (75%, 15/20), β_2_-microglobulin (100%, 10/10), C-reactive protein (82%, 14/17), and ferritin (88%, 7/8) and the erythrocyte sedimentation rate (83%, 10/12) were elevated. Bilateral involvement was seen in 21 of 26 patients (81%); 12 of 19 evaluated patients with bilateral lesions (63%) were confirmed to have adrenal insufficiency. On computed tomography (CT), the mean tumor diameter was 7.31 ± 3.35 cm and the median Hounsfield density was 37.0 HU (range: 31.0–45.0 HU); 67% (10/15) and 27% (4/15) of lesions presented with mild and moderate enhancement after injection of contrast medium. ^18^F-fluorodeoxyglucose positron emission tomography (FDG PET)-CT revealed not only an adrenal tumor but also extra-adrenal lesions. Diffuse large B-cell lymphoma (DLBCL) was the most common phenotype (92%, 24/26). Ninety-two percent (24/26) of patients received chemotherapy while 8% (2/26) received unilateral adrenalectomy plus chemotherapy. The prognosis of PAL was poor, with a general survival time of 7.20 ± 5.18 months.

**Conclusion:**

PAL is a rare disease. The clinical characteristics of PAL include loss of appetite and weight loss. Endocrine evaluation should be performed to determine whether patients have adrenal insufficiency, especially patients with bilateral lesions. FDG-PET appears to be more accurate than other imaging modalities in revealing extra-adrenal sites. Better therapy is required to improve the poor prognosis of PAL.

## Introduction

Most lymphomas originate from lymph nodes, while up to a quarter develop from extra-lymph node lymphoid or non-lymphoid tissue. Primary extranodal lymphoma (PEL) has been reported to account for 25%–40% of all cases of lymphoma in Western countries (1) and 45.9% in Taiwan respectively (2). The most commonly involved sites in PEL are the gastrointestinal tract, central nervous system, and skin (3). Lymphoma originating from the endocrine system accounts for 3% of cases of PEL, and primary adrenal lymphoma (PAL) accounts for only 0.2% of those lymphomas (4). There are only 250 cases described in the English-language literature worldwide to date (5); the majority of published articles about PAL are case reports or case-series studies with only a limited number of patients. The common features of PAL are male sex, older age, bilateral lesions, adrenal insufficiency, and poor outcomes, based on data mostly from Western countries (6). Due to the extreme rarity of this disorder, difficulty in differential diagnosis such as adrenocortical carcinoma or pheochromocytoma, and the lack of guidelines, physicians may face the difficulty in diagnosing and treating PAL.

The objective of this study is to summarize the clinical features, biological and imaging characteristics, and outcomes of 26 patients with PAL in the northern and southern regions of China in order to characterize this entity, which may improve the differential diagnosis of adrenal masses and finally improve prognosis through early diagnosis and treatment.

## Materials and Methods

Nineteen and seven patients with PAL were diagnosed at the Chinese People’s Liberation Army General Hospital in Beijing and the First Affiliated Hospital of Xiamen University in Xiamen, respectively, from July 2007 to July 2017. The diagnostic criteria for PAL were as follows (5): (a) histologically proven lymphoma that involves at least one adrenal gland; (b) no prior history of lymphoma; and (c) adrenal lesions are unequivocally dominant if lymph nodes or other associated organs involved. The demographics, clinical manifestations, biochemical examination, imaging features, pathological type, and prognosis of 26 patients with PAL were retrospectively reviewed. The diagnosis of adrenal insufficiency (AI) was based on an 8 am cortisol value <5 µg/dl in combination with a high adrenocorticotropic hormone level.

Statistical analyses were performed using the IBM Statistical Package for the Social Sciences version 16.0. Mean ± standard deviation (SD) and median (interquartile range) are used to describe variables with normal and non-normal distributions, respectively. Frequency and percentage are used to describe categorical data. Group variables with a normal distribution were compared using the t-test, while categorical data were compared using the *χ* ^2^ test.

## Results

### Baseline Demographic

**Figure 1:**
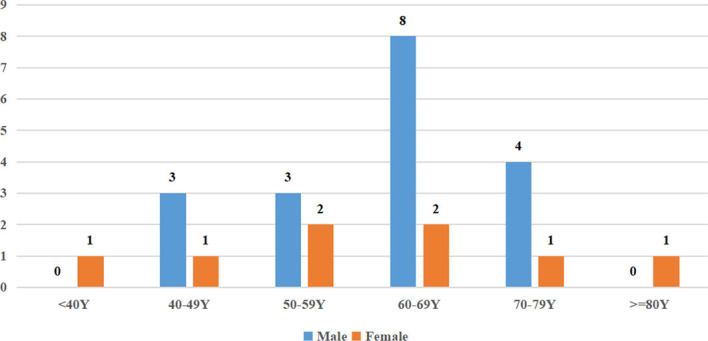
Age distribution of PAL in this series.

A total of 26 cases of PAL were identified (clinical characteristics are summarized in **Table 1**), which included 18 male (69%) and 8 female (31%) patients, with a sex ratio of 2.25:1. The mean age of the patients at diagnosis was 60.84 ± 13.14 years. The distribution of patients according to age is illustrated in **Figure 1**, and PAL was most commonly seen in patients aged 60–69 years. The mean body mass index (BMI) at diagnosis across all patients was 23.26 ± 3.92 kg/m^2^. None of our patients had a history of autoimmune disease, other malignancy, or immune suppression therapy. In our cohort, the largest number of patients were diagnosed in the Endocrinology Department (12/26, 46%), followed by the Hematology Department (4/26, 15%), Urology Department (4/26, 15%), Gastroenterology Department (4/26, 15%), and Respiratory Department (2/26, 8%).

**Table 1:** Clinical characteristics of the study population.

	Total
n	26
Age (years)	60.84 ± 13.14
Sex (male/female)	18/8
BMI (kg/m^2^)	23.26 ± 3.92
Autoimmune disease (n)	0
Immune suppression (n)	0
Malignances (n)	0
Elevated LDH (n, %)	15, 75%
Elevated CRP (n, %)	14, 82%
Elevated ESR (n, %)	10, 83%
Elevated β2-MG (n, %)	10, 100%
Elevated ferritin (n, %)	7, 88%
Adrenal insufficiency (n, %)	12, 63%
Unilateral/bilateral involvement (n)	5/21
Tumor size (cm)	7.31 ± 3.35
CT value (HU)	37.0
Non-enhancement (n, %)	1, 6%
Mild-enhancement (n, %)	10, 67%
Moderate-enhancement (n, %)	4, 27%
Homogeneous enhancement (n, %)	10, 71%
Heterogeneous enhancement (n, %)	4, 29%
Mean SUV of adrenal lesion	17.72 ± 8.64
Mean SUV of extra-adrenal lesion	11.82 ± 6.08
Diffuse large B cell lymphoma/extranodal NK/T cell lymphoma (n)	24/2

### Clinical Manifestations

**Figure 2:**
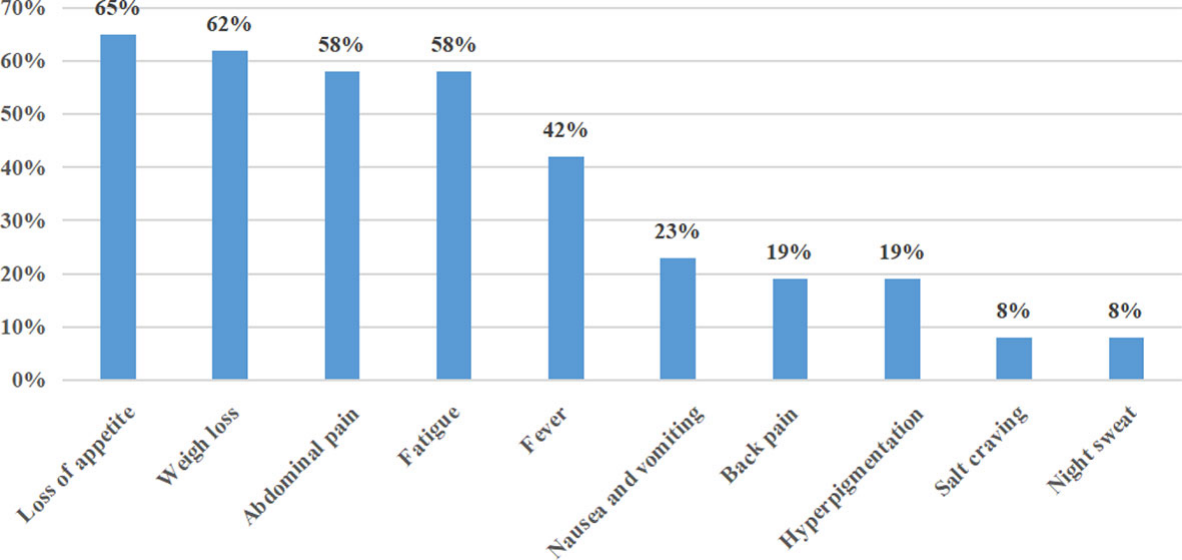
Frequency of clinical presentations of PAL in this series.

The duration of disease ranged from 0.46 to 3.00 months, with a median duration of symptoms of 1.0 month. At the time of initial diagnosis, the most common presenting symptoms were loss of appetite (17/26, 65%), weight loss (16/26, 62%), abdominal pain (15/26, 58%), and fatigue (15/26, 58%). Less common presenting symptoms included fever (11/26, 42%), nausea and vomiting (6/23, 23%), back pain (5/26, 19%), hyperpigmentation (5/26, 19%), salt craving (2/26, 8%), and night sweats (2/26, 8%) (**Figure 2**). The initial presenting symptom in most cases (13/26, 50%) was abdominal pain. On physical examination, posture hypotension, lymphadenopathy, abdominal masses, and hepatosplenomegaly were present in 23% (6/26), 15% (4/26), 4% (1/26), and 4% (1/26) of our patients, respectively.

### Biological Profile

The level of lactate dehydrogenase (LDH) was elevated in 15 (75%) out of the 20 patients in whom it was tested, with a mean value of 493.94 ± 382.63 U/l (40–250 U/l). Serum β_2_-microglobulin (β_2_-MG) was elevated in all 10 tested patients (100%), with a mean value of 0.40 ± 0.18 mg/dl (0.07–0.18 mg/dl). C-reactive protein (CRP) was elevated in 82% of patients (14/17) with a mean value of 3.66 ± 3.53 mg/dl (0–0.8 mg/dl). Twelve patients underwent erythrocyte sedimentation rate (ESR) tests, and 10 patients (83%) had elevated values, with a mean value of 42.17 ± 23.49 mm/h (0–20 mm/h). Plasma ferritin levels were elevated in seven of eight tested patients, with a mean value of 1,005.00 ± 756.45 ng/ml (30–400 ng/ml). Twelve of the 19 (63%) evaluated patients had AI, and all cases of AI were caused by bilateral lymphomatous involvement.

### Imaging Features

**Figure 3:**
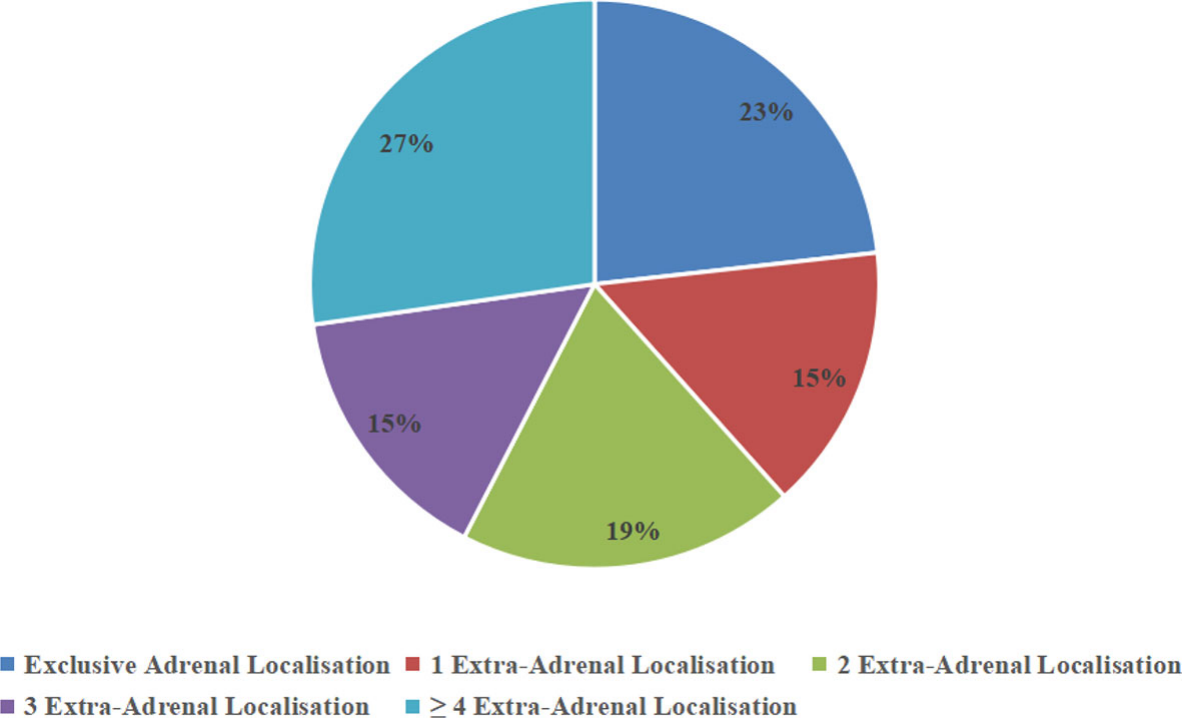
Percentage of adrenal and extra-adrenal locations.

Bilateral adrenal gland involvement was seen in 81% (n = 21) of cases. Among five patients with unilateral disease, the left–right ratio was 3:2. Six patients had only adrenal lesions with disease in no other locations. Four patients had involvement at one extra-adrenal site, five patients had involvement at two extra-adrenal sites, four patients had involvement at three extra-adrenal sites, and seven patients had involvement at four or more sites (**Figure 3**). The diameter of the tumors was 7.31 ± 3.35 cm.

### Computed Tomography

**Figure 4:**
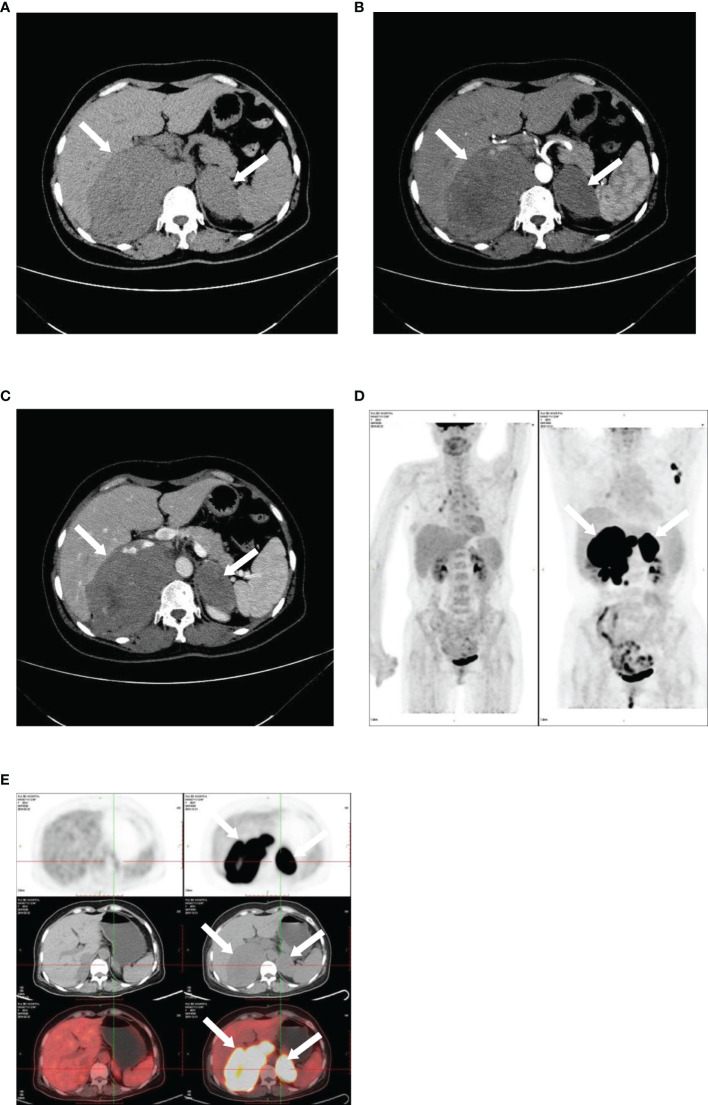
Imaging of bilateral primary adrenal lymphoma. CT and PET images illustrating bilateral primary adrenal lymphoma. CT scans: **(A)**, non-contrast phase; **(B)**, arterial phase; **(C)**, venous phase. 18F-FDG PET-CT: **(D, E)**.

On axial images, the proportion of PAL with a regular pattern was 68% (27/40) and 32% (13/40) with an irregular pattern. The frequency of well-defined and ill-defined tumors was 63% (25/40) and 37% (15/40), respectively. Non-contrast and contrast-enhanced computed tomography (CT) was performed in 26 and 8 cases, respectively. The median Hounsfield density was 37.0 Hounsfield units (HU) (range: 31.00–45.00 HU). After intravenous injection of contrast medium, 7% (1/15) of lesions showed no enhancement, 67% (10/15) of tumors showed mild enhancement, and 27% (4/15) of lesions showed moderate enhancement. Homogeneous and heterogeneous enhancement on CT was seen in 71% (10/14) and 29% (4/14) of tumors, respectively (**Figure 4**).

### ^18^F-Fluorodeoxyglucose Positron Emission Tomography-Computed Tomography

Twenty-one patients underwent 3.6 18F-fluorodeoxyglucose positron emission tomography-computed tomography (^18^F-FDG PET-CT) examination in our study. The standard uptake value (SUV) of the adrenal gland was elevated in all patients, with a mean value of 17.72 ± 8.64, while mean SUV of ex-adrenal lesions was 11.82 ± 6.08. The uptake of ^18^F-FDG in the adrenal gland was more intense than the uptake in extra-adrenal lesions. Fourteen patients had lymph node involvement, with abdominal lymph node involvement being the most common (93%, 13/14), followed by the mediastinal (50%, 7/14) and neck (29%, 4/14) involvement. There were 17 cases of extranodal. The most frequently affected locations were the bone (47%, 8/17), liver (29%, 5/17), and lung (24%, 4/17) (**Table 2**).

**Table 2:** Summary of extra-adrenal infiltrations.

Affected lymph nodes	Cervical	4
	Axillary	1
	Mediastinal	7
	Abdominal	13
Affected organ/tissues	Bone	8
	Liver	5
	Lung	4
	Spleen	3
	Kidney	3
	Testicle	2
	Pancreas	1
	Colon	1
	Meningeal	1

### Histology

In 92% (24/26) of patients, the diagnosis was based on histopathological examination and immunohistochemistry of the adrenal tissue following adrenal biopsy. Only 8% (2/26) of patients underwent initial unilateral adrenalectomy. The most frequent pathology was diffuse large B-cell lymphoma (DLBCL) in 92% (24/26) of patients, while the histology in 8% (2/26) of patients was extranodal NK/T cell lymphoma.

### Treatment and Prognosis

Twenty patients with DLBCL received treatment with prednisone (90 mg, days 1–5), vincristine (1.4 mg/m^2^ day 1), cyclophosphamide (750 mg/m^2^ day 1), or doxorubicin (45 mg/m^2^, day 1) along with rituximab (375 mg/m^2^, day 0) (R-CHOP regimen). Two patients with extranodal NK/T cell lymphoma received methotrexate (3 g, day 1), heterocyclic phosphamide (3 g, day 2; 2 g, day 3–4), dexamethasone (40 mg, days 2–4), etoposide (100 mg, days 2–4), and asparaginase (10,000 U; days 8, 10, and 13) (SIMLE regimen). To date, twenty-two patients have died till now, with a mean survival time of 7.20 ± 5.18 months; four patients were lost during the follow-up.

## Discussion

We discussed the demographic findings, clinical presentation, biological evaluations, imaging characteristics, pathology, treatment, and prognosis of PAL in two centers from China during the last 10 years. To our best knowledge, the current study is the only reported double center experience of PAL in China.

PAL usually develops in elderly male patients, with a median age at presentation ranging from 48 to 68 years and male/female ratio of 1.8:1–7:1 (5–9); our findings were consistent with the reported age and sex ratio ranges. Dobrinja et al. suggest that the highest incidence occurs in adults approximately 60 years old (10), which corresponds with the present series. The BMI was similar to the existing literatures noted by Yumi, Horiguchi and Huang (11–13), while it was lower than that reported by Laurent et al. (6). This may be attributed to the difference of ethnic, diet structure, and environment.

The median time from symptom onset to the initial diagnosis of PAL was 1 month, which is slightly shorter than that reported for Asian cases (median time, 1.5 months; range, 0.25–12 months) (14–24). Since the adrenal glands are concealed in the retroperitoneum and endocrine inactivity, the initial signs and symptoms are often unspecific. As established by findings from a meta-analysis (5), the most common symptoms were B-symptoms, which include fever, weight loss, night sweats (68%), pain (42%), and fatigue (36%), which corresponds with our data. Regarding signs, adenopathy, splenomegaly, and hepatomegaly were present in 18%, 14%, and 11% in a cases series from Laurent et al. (6), which was slightly higher than the rates in our case series (15%, 4%, and 4%). More specific symptoms and signs, such as hyperpigmentation (41%–74%), salt craving (38%–74%), and postural hypotension (55%–58%), were related to AI in a previous review (25); these signs were present in 42%, 25%, and 50%, respectively, in our population, suggesting that there were few endocrinology consultations or not all patients were managed by the Endocrinology Department. In addition, AI often presented in patients with bilateral adrenal involvement. In the 12 confirmed cases of AI in 19 assessed patients, no patient with unilateral involvement had AI, which is in accordance with the principle that >90% adrenal parenchymal destruction will lead to AI given the huge reserve in adrenal function (26). Consequently, endocrinology evaluation should be performed in patients with bilateral adrenal involvement and/or clinical symptoms or signs of AI. Immune system dysfunction has been proposed as an etiology of PAL (27). Wang et al. reviewed 55 patients with PAL and found that 13% had autoimmune disease (28). However, none of our patients had any prior history of carcinoma or autoimmune disease, which is in accordance with the literatures noted by Kasaliwal and Rashidi (5, 9).

In our series, 75% (15/20) of patients had increased LDH levels, which is in accordance with data shown by Laurent et al. (74%, 20/27) (6), indicating that tumor burden increases with cell turnover but lower than that reported by Rashidi et al. (88%, 70/80) (5), which may be due to the small sample size in the current series. Elevated serum β_2_-MG levels may indicate hematological disease, which was present in 100% (10/10) of our cases, higher than the incidence reported by Laurent el al. (71%, 15/21) (6). In addition, serum β_2_-MG and CRP levels were the most important predictive factors for overall survival in DLBCL (29–31), which explains the lower median survival in our study compared to that noted by Laurent et al. (6). The ESR and ferritin levels may indicate an inflammatory syndrome, but neither of them lead to a diagnosis of DLBCL or correlate with prognosis.

Diagnostic imaging includes CT, magnetic resonance imaging (MRI), and PET-CT. CT is considered to be the most important imaging modality in evaluating adrenal masses as it can be used for their localization, visualization, and characterization. On CT, 81% of patients (21/26) had bilateral involvement, which was a slightly higher rate than that reported in previous related studies (32); the delayed diagnosis in our series may reflect that unilateral involvement is the process of bilateral lesions. The mean tumor diameter at the time of diagnosis in our case series was 7.31 ± 3.35 cm, which is similar to that in previous related studies (33). The Cleveland Clinic performed a large retrospective study including 299 cases and determined a threshold of 10 HU on non-enhanced CT to distinguish benign and malignant adrenal masses, with a sensitivity and specificity of 79% and 96%, respectively (34). In our series, the median attenuation of PAL was 37 HU, which is in accordance with the density reported by Zhou et al. (35). In addition, the dominant pattern of enhancement after intravenous injection of the medium showed that 88% of our patients had a “slight to moderate enhancement pattern”, which was reported to occur in 78%–100% of cases in previous reports (5, 35), given that PAL is not a hypervascular tumor. This pattern is different from adrenocortical carcinoma and pheochromocytoma, which are hypervascular lesions and show significant enhancement. Furthermore, 71% of patients displayed “homogeneous enhancement.” Conversely, previous studies showed that PAL tends to exhibit a heterogeneous enhancement pattern (36), whereas a study that included 28 cases demonstrated that most had homogeneous or slightly inhomogeneous enhancement (32). ^18^F-FDG PET-CT provides anatomical and functional information, both in the adrenal gland and in other involved sites. Twenty-one patients underwent ^18^F-FDG PET-CT, which makes our study the largest collection of patients with PAL examined using PET-CT. Due to the metabolic hyperactivity in PAL, SUV levels were elevated with a mean value of 17.72 ± 8.64 and 11.82± 6.08 in adrenal and extra-adrenal lesions, which is slightly lower than the values in a previous report (6). In addition, compared with CT and MRI, PET-CT provides more precise evaluation of PAL extension. Eighty-one percent (17/21) of our cases exhibited ex-adrenal extension on PET-CT, which is higher than the rate reported in the study by Laurent, consistent with the aggressive biological characteristics of PAL.

While clinical manifestations, biological, and imaging can be helpful, a definitive diagnosis can only be made histologically using tissue obtained *via* needle core biopsy, incisional or excisional biopsy, or autopsy. The DLBCL phenotype was predominant (92%) in our series and ranges 78%–94% in previous reports (12, 33, 37). PAL has a poor prognosis. Two decades ago, the median survival was 4 months (38), with the longest reported survival of 15 months (39). Recently, with the use of the R-CHOP regimen instead of the CHOP regimen, the clinical outcome has improved (40). Unfortunately, the mean survival in our case series was 7.20 ± 5.18 months, which was worse than that in earlier studies (40), suggesting hidden manifestation and that patients may have been diagnosed at a late stage of disease.

## Conclusion

PAL is a rare entity that usually occurs in elderly men and more commonly presents with bilateral lesions. ^18^F-FDG PET-CT is a major tool in the diagnosis of adrenal lesions and extra-adrenal extensions. The dominant histological phenotype is DLBCL, which can lead to adrenal insufficiency, and has a poor prognosis. Better therapy is required to improve outcomes.

## Data Availability Statement

The original contributions presented in the study are included in the article/supplementary material. Further inquiries can be directed to the corresponding author.

## Ethics Statement

The studies involving human participants were reviewed and approved by the Ethics Committee of West China Hospital of Sichuan University (No. 2019-229). The patients/participants provided their written informed consent to participate in this study. Written informed consent was obtained from the individual(s) for the publication of any potentially identifiable images or data included in this article.

## Author Contributions

JZ and FY: collected the data and wrote the manuscript draft. YC, LZ, KC, ZL, JD, YM, and ML: contributed to the discussion and revision. GY: designed the study and revised the submission. All authors contributed to the article and approved the submitted version.

## Conflict of Interest

The authors declare that the research was conducted in the absence of any commercial or financial relationships that could be construed as a potential conflict of interest.

## Publisher’s Note

All claims expressed in this article are solely those of the authors and do not necessarily represent those of their affiliated organizations, or those of the publisher, the editors and the reviewers. Any product that may be evaluated in this article, or claim that may be made by its manufacturer, is not guaranteed or endorsed by the publisher.
